# Time-Resolved Inspection of Ionizable Lipid-Facilitated Lipid Nanoparticle Disintegration and Cargo Release at an Early Endosomal Membrane Mimic

**DOI:** 10.1021/acsnano.4c04519

**Published:** 2024-08-12

**Authors:** Nima Aliakbarinodehi, Simon Niederkofler, Gustav Emilsson, Petteri Parkkila, Erik Olsén, Yujia Jing, Mattias Sjöberg, Björn Agnarsson, Lennart Lindfors, Fredrik Höök

**Affiliations:** † Department of Physics, Division of Nano and Biophysics, Chalmers University of Technology, Göteborg 41296, Sweden; ‡ Advanced Drug Delivery, Pharmaceutical Sciences, AstraZeneca R&D, Mölndal 43181, Sweden

**Keywords:** lipid nanoparticle (LNP), mRNA delivery, endosomal escape, early endosomal membrane mimic, lipid nanoparticle fusion

## Abstract

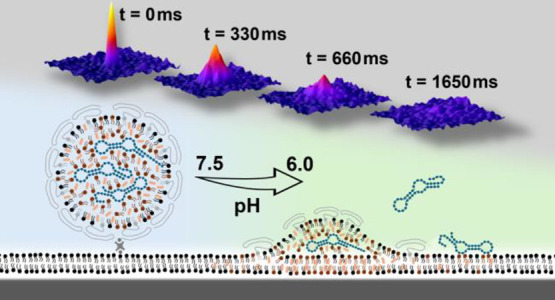

Advances in lipid nanoparticle (LNP) design have contributed notably to the emergence of the current clinically approved mRNA-based vaccines and are of high relevance for delivering mRNA to combat diseases where therapeutic alternatives are sparse. LNP-assisted mRNA delivery utilizes ionizable lipid-mediated cargo translocation across the endosomal membrane driven by the acidification of the endosomal environment. However, this process occurs at a low efficiency, a few percent at the best. Utilizing surface-sensitive fluorescence microscopy with a single LNP and mRNA resolution, we have investigated pH-controlled interactions between individual LNPs and a planar anionic supported lipid bilayer (SLB) formed on nanoporous silica, mimicking the electrostatic conditions of the early endosomal membrane. For LNPs with an average diameter of 140 nm, fusion with the anionic SLB preferentially occurred when the pH was reduced from 6.6 to 6.0. Furthermore, there was a delay in the onset of LNP fusion after the pH drop, and upon fusion, a significant fraction (>70%) of mRNA was released into the acidic solution representing the endosomal lumen, while a fraction of mRNA remained bound to the SLB even after reversing the pH to neutral cytosolic conditions. Finally, a comparison of the fusion efficiency of two LNP formulations with different surface concentrations of gel-forming lipids correlated with differences in the protein translation efficiency previously observed in human primary cell transfection studies. Together, these findings emphasize the relevance of biophysical investigations of ionizable lipid-containing LNP-assisted mRNA delivery mechanisms while potentially also offering means to optimize the design of LNPs with enhanced endosomal escape capabilities.

## Introduction

In mRNA therapeutics, endogenous cellular machineries are utilized to produce therapeutic proteins, thereby providing a promising means to treat a multitude of diseases where conventional medication strategies fail.^1,2^ To overcome the inherent instability of mRNA and its low capacity to be naturally taken up by cells, a large number of viral and synthetic therapeutic vectors have been developed.^3−5^ Despite high transfection efficacy,^6^ viral vectors are associated with challenges related to genetic interference, low cargo payload, toxicity, and immunogenicity.^2,7,8^ This, in turn, has spurred intense efforts in designing nonviral mRNA vectors, as recently manifested by the lipid nanoparticle (LNP)-based COVID-19 vaccines developed by Pfizer/BioNTech^9^ and Moderna.^10^

The most efficient LNPs designed for mRNA delivery are formulated using ionizable lipids together with a set of helper lipids, typically cholesterol, gel-phase forming phospholipids, and polyethylene glycol (PEG)-modified lipids.^11^ Efficient mRNA encapsulation, appropriate LNP structures, and desired stability^12,13^ are typically obtained by tuning the ratio between mRNA and lipid components utilizing microfluidic-assisted rapid mixing precipitation protocols.^14^ This approach has been demonstrated to generate LNPs that display successful endocytic uptake accompanied by mRNA-assisted protein expression, low clearance and degradation, and even specific cell targeting.^4,15^ However, LNPs offer significantly lower transfection efficacy than their viral counterparts,^6^ which is attributed to both extra- and intracellular obstacles, of which the endosomal escape event has been identified as a key bottleneck.^16,17^

This process, during which mRNA is translocated across the endosomal membrane into the cytosol of the target cell, depends on the gradual acidification of the endosomal environment,^18,19^ which in turn is believed to promote electrostatic attraction between the cationic ionizable lipid contained in LNPs and the anionic endosomal membranes. In the case of mRNA, it was recently shown that LNP disintegration and subsequent mRNA translocation across the endosomal membrane preferentially occur in early endosomes,^20^ in which the charge of the endosomal membrane is primarily controlled by phosphatidylserine (PS) lipids.^21^ However, even if cellular endocytic LNP uptake is usually very efficient, functional mRNA delivery is not; in fact, with ionizable lipid-containing LNPs designed for siRNA delivery, less than 2% of the endocytosed cargo resulted in a functional response,^11,22^ and the efficacy is even lower in the case of high molecular-weight mRNA.^23^

Insights of this type are typically obtained using advanced optical imaging approaches utilizing in vitro cellular assays,^6,17,23,24^ which are further corroborated through in vivo studies.^25^ Recent work has shown that fundamental mechanistic insights with respect to the nature of LNP interactions with cellular membranes can also be gained by making use of simplified mimics of the anionic endosomal membrane. For example, pH-induced binding of LNPs to anionic lipid monolayers formed at an air–water interface revealed that pH-induced lipid transfer induces structural alterations of endosomal membrane mimics.^26^ By forming a supported lipid bilayer (SLB) containing 6 mol % POPS on a planar glass substrate, thus mimicking the anionic character of the early endosomal membrane, it was shown that pH-induced electrostatically controlled LNP binding to the membrane is accompanied by ionizable lipid transfer that leads to charge neutralization of the anionic SLB, presumably relevant in the context of endosomal arrest.^27^

In this work, we have used time-resolved dual-color total internal reflection fluorescence (TIRF) microscopy to investigate pH-induced interactions between individual LNPs and an anionic SLB. Inspired by previous work demonstrating that investigations of membrane-enveloped-virus fusion benefit from minimizing the contact between the SLB and the underlying support,^28,29^ the anionic SLB was formed on a porous silica substrate,^30,31^ previously shown to display significantly higher lipid diffusivity than when formed on planar glass, and also to be compatible with lipid molecular translocation across the lipid membrane.^32^ Since cellular endocytic LNP uptake is believed to be mediated by the specific binding between ApoE spontaneously adsorbed on the LNP surface in the presence of serum proteins and LDL receptors present on the surface of recipient cells,^33,34^ LNPs are expected to reside in close proximity to the endosomal membrane. The LNPs were, therefore, molecularly anchored to the anionic SLB using a NeutrAvidin–biotin linkage, which also enabled continuous time-resolved imaging of LNPs during the gradual endosomal acidification process to be simulated by varying the pH of the bulk solution by microfluidic-assisted liquid exchange.

The investigation was primarily focused on a particular LNP formulation containing DLin-MC3-DMA as the ionizable lipid, and cholesterol, DSPC, and DMPE-PEG2000 serving as helper lipids, which in previous in vitro cellular assays was demonstrated to display efficient cellular uptake and high protein expression levels.^35^ The LNP fusion kinetics and the fate of the LNP cargo were microscopically visualized by staining the LNPs with 0.06 mol % lissamine rhodamine B-labeled DOPE (Rhod-DOPE) and with 20% of the mRNA being Cy5-labeled (Cy5-mRNA). Individual fusion events were statistically analyzed with respect to (i) Rhod-DOPE and Cy5-mRNA release kinetics, (ii) the pH-dependency of the diffusivity of individual mRNA and mRNA clusters that remained attached to the anionic SLB after completed LNP fusion, and (iii) the wait time observed between the rapid (<1 s) pH drop and the actual onset of LNP fusion with the anionic SLB. The LNP fusion efficiency was also compared with an additional LNP formulation, designed to display a two times higher surface concentration of the gel-phase forming DSPC lipid, a variation previously shown to display similar cellular uptake efficiency, but more than 1 order of magnitude lower protein expression levels.^35^ The mechanistic insights related to pH-induced LNP disintegration revealed through this investigation are discussed in the context of possible endosomal escape pathways and how the use of simplified biomimetic assays may help advance the design of more efficient mRNA delivery vectors.

## Results and Discussion

### The pH Dependence of the LNP Fusion Efficiency

The anionic SLB was formed on nanoporous silica with pore dimensions of around 6 nm through lipid vesicle adsorption-induced SLB formation^30^ using lipid vesicles composed of 93.5 mol % POPC and 6 mol % POPS, representing the negative charge of the early endosomal membrane.^21^ To enable visualization of the SLB formation process using TIRF microscopy and to perform lateral mobility determinations using fluorescence recovery after photobleaching (FRAP),^36^ the lipid vesicles contained 0.5 mol % NBD-labeled lipids (NBD-DOPE). The TIRF and FRAP analyses revealed the successful formation of a continuous SLB with a diffusivity constant *D* of 4.4 ± 0.3 μm^2^ s^–1^ (*n* > 3) and an immobile fraction of 0.06 ± 0.02 (*n* > 3). This is at least twice the diffusivity typically obtained for SLBs with the same lipid composition formed on planar glass,^27^ attributed to reduced lipid pinning at the interface between the SLB and the nanoporous silica support. It is also worth noting that the reduced contact area of an SLB formed on nanoporous silica, compared to planar silica, is expected to decrease electrostatic repulsion between the substrate and negative lipids in the SLB, thus reducing the risk for the asymmetric distribution of POPS between the two bilayer leaflets.

**Figure 1:**
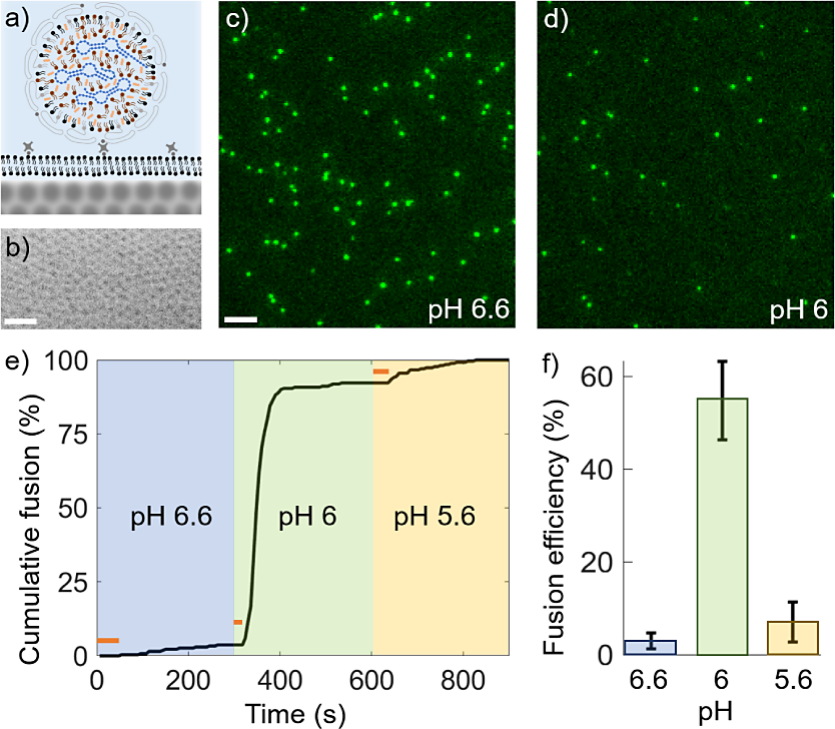
LNP fusion efficiency versus pH. a) Schematic illustration of a biotin-modified LNP bound via NeutrAvidin linking to a planar biotin-lipid-containing mimic of the early endosomal membrane formed on a nanoporous silica substrate. b) SEM image showing the estimated 6 nm pore size of the porous silica substrate (scale bar = 50 nm). c, d) TIRF micrographs of tethered LNPs (Rhod-DOPE emission) 5 min after exposure to c) pH 6.6 (scale bar = 5 μm) and d) pH 6.0. e) Cumulative number of fusion events displayed in the percentage of total fusion events versus time upon subsequent reductions in pH from 7.5 to 6.6, 6.0, and 5.6. The orange bars indicate the wait time between the reduction in pH and the first recorded fusion event. f) Fusion efficiency versus pH displayed in relation to the total number of initially tethered LNPs.

To enable time-resolved TIRF imaging of individual LNP fusion events, LNPs with a number-average diameter and polydispersity index (PDI) of 140 nm and <0.1, respectively, were formulated using 53.47 mol % ionizable cationic lipids (DLin-MC3-DMA), 4.65 mol % DSPC, 41.114 mol % cholesterol, 0.7 mol % PEG-modified lipids (DMPE-PEG2000), 0.06 mol % fluorescent rhodamine-labeled lipids (Rhod-DOPE), and 0.006 mol % biotin-modified lipids (DSPE-PEG2000-Biotin), while the eGFP-encoding mRNA cargo was composed of Cy5-labeled mRNA (Cy5-mRNA) and nonlabeled mRNA at a 1:4 ratio (Material and Methods, and Figure S1 and Table S1). The LNPs were bound using NeutrAvidin as a linker between biotin-PEG-lipids in the LNPs (∼70 per LNP) and 0.05 mol % of Biotin-Cap-DOPE in the SLB, as schematically depicted in Figure 1a.

Binding of biotin-modified LNPs to the NeutrAvidin-modified anionic SLB formed on nanoporous silica (Figure 1b) was recorded using dual-color TIRF microscopy at 3 frames per second (fps) upon LNP injection at a concentration of ∼10^9^ LNPs/mL in a flow cell (3.8 × 17 × 0.4 mm in width × length × height) at a volumetric flow rate of 140 μL min^–1^ until an LNP coverage of ∼0.03 μm^–2^ (∼600 LNPs per field of view) was reached, typically within 5 to 10 min. After terminating the LNP binding by rinsing the channel with a buffer solution at pH 7.5, the pH of the flowing solution was subsequently changed via rapid liquid exchange (<1 s within the field of view) from 7.5 to 6.6 (Movie S1), 6.0 (Movie S2), and 5.6 (Movie S3), while continuously recording LNP fluorescence emission for around 5 min at each pH value.

The most dramatic response was observed upon reducing the pH from 6.6 to 6.0, resulting in a drastic decrease in the number of LNPs with a detectable Rhod-DOPE emission signal, as illustrated in Figure 1c,d. A cumulative sum of all individual fusion events versus time upon the subsequent pH reduction steps (Figure 1e) shows that very few fusion events are detected when the pH is reduced from 7.5 to 6.6, while most fusion events occur after a wait time of ∼10 s and within less than 100 s upon the reduction of the pH from 6.6 to 6.0. Inspection of the micrographs revealed fusion efficiencies relative to the total number of bound LNPs at pH 7.5 of around 3, 54, and 10% at pH 6.6, 6.0, and 5.6, respectively (Figure 1f).

To estimate the degree of ionization of the LNPs, that is, the process responsible for inducing electrostatic attraction to the anionic membrane, the fraction of ionized DLin-MC3-DMA as a function of pH was measured using anionic fluorescent dye 2-(*p*-toluidino)-6-naphthalene sulfonic acid (TNS), which undergoes significant fluorescent enhancement when binding to positively charged lipids.^37,38^ The TNS assay displays a relatively sharp transition around an inflection point at pH 6.6, with 20 and 80% of DLin-MC3-DMA available for TNS binding being ionized at around pH 7.5 and 6.0, respectively (Figure S2). While it remains uncertain whether nonsurface-exposed ionized DLin-MC3-DMA is available for TNS binding, these results suggest that substantial ionization of DLin-MC3-DMA is required to initiate electrostatically driven fusion between ionized LNPs and the anionic SLB.

### The Spatiotemporal Dynamics Differ between Lipids and the Cargo upon LNP Fusion

**Figure 2:**
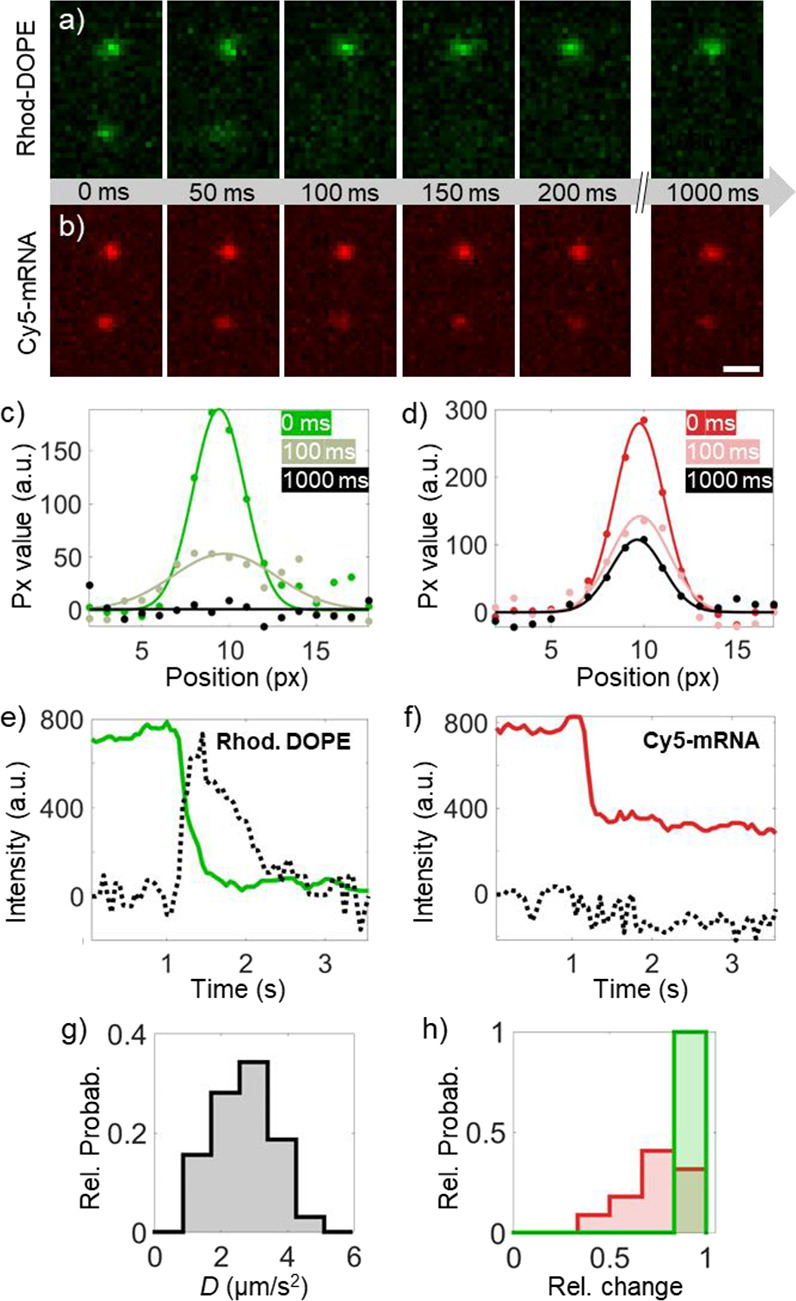
Time-resolved inspection of pH-induced LNP fusion and cargo escape. TIRF micrographs showing a) Rhod-DOPE and b) Cy5-mRNA emission signals for two tethered LNPs measured at 20 fps upon a reduction in pH from 7.5 to 5.6 (scale bar = 1 μm). The upper LNP does not show any response to the pH change in this time interval while the lower does. Background-subtracted spatiotemporal emission profiles for individual LNPs were fitted to two-dimensional Gaussian profiles that are shown in c) and d) represented as one-dimensional averages for Rhod-DOPE and Cy5-mRNA, respectively. These were further used to display the temporal evolution of the total LNP emission intensity (solid lines) and the corresponding emission from and area surrounding the LNP docking site (dashed lines) for e) Rhod-DOPE and f) Cy5-mRNA. g) Diffusion constants, *D,* obtained from the variance, σ^2^ = 2*Dt,* of the time evolution of the two-dimensional Gaussian emission profiles from 32 LNPs.^40^ h) Relative change  in the Rhod-DOPE (green) and Cy5-mRNA (red) emission intensities for LNPs that undergo pH-induced fusion.

The temporal evolution of the LNP fluorescence emission signal upon the actual fusion event is characterized by a synchronized reduction in both Rhod-DOPE and Cy5-mRNA emission signals (lower LNP in Figure 2a,b). However, the amount of signal reduction differs significantly, and from analyzing a two-dimensional Gaussian function fitted to the background-subtracted emission profile of individual LNPs (Figure 2c,d), as previously described for the analysis of single lipid vesicle fusion events,^29^ significant differences in the spatiotemporal evolutions for the two fluorescent signals are revealed (Figure 2e,f).

Considering the Rhod-DOPE emission signal first, it displays a gradual reduction on the time scale on the order of a few hundred milliseconds (green curve in Figure 2e). This decrease is accompanied by a concurrent increase in the Rhod-DOPE emission intensity in the area surrounding the docking site of the LNP (dashed curve in Figure 2e). The time evolution of the variance, σ^2^, of the fitted Gaussian function reveals a mean diffusion coefficient *D* for Rhod-DOPE of 2.6 ± 0.9 μm^2^ s^–1^ (Figure 2g), and a reduction in the total emission intensity by more than 90% (Figure 2h). These findings are indicative of near-complete escape of Rhod-DOPE into the anionic SLB, in analogy with pH-induced fusion of enveloped viruses to membrane mimics when visualized using dye-labeled lipids.^28^ The somewhat lower diffusivity of Rhod-DOPE compared to the unperturbed anionic SLB of ∼4.4 μm^2^ s^–1^ is attributed to the nature of the lipid constituents of the LNP, including DLin-MC3-DMA, DSPC, and cholesterol, the latter of which is known to reduce membrane mobility.

In contrast, the Cy5-mRNA emission signal displays a rather abrupt drop (<300 ms) upon fusion, resulting in an intensity reduction of around 70% (red curve in Figure 2h), but unlike the dye-labeled Rhod-DOPE lipid, the Cy5-mRNA signal reveals no detectable signs of a concurrent increase in the area around the docking site of the LNP (dashed line in Figure 2f). This suggests that the measured response is caused either by (i) mRNA translocation across the anionic SLB into the porous regions of the substrate, where the illumination intensity is approximately four times lower than the TIR illumination intensity at the nanoporous interface,^39^ or (ii) photophysical changes of the Cy5 emission induced upon LNP collapse, or (iii) Cy5-mRNA release into the acidic bulk solution above the anionic SLB,^40^ or a combination of these processes.

Inspection of fusion kinetics by employing epi-illumination, which ensures consistent illumination across the nanoporous interface, revealed similar kinetics (Figure S3) to that shown in Figure 2f, with no apparent indication of Cy5-mRNA being translocated across the membrane and into the nanoporous substrate, suggesting that this process is most likely not the predominant contribution to the observed reduction in the fluorescence intensity. Considering photophysical changes, it should be noted that each Cy5-mRNA contains on average ∼34 Cy5 dyes per mRNA (see Materials and Methods). With 20% of the mRNA cargo being labeled, and with on average 200 mRNA per 140 nm diameter LNP,^35^ the mean distance between Cy5 dyes within an LNP becomes ∼10 nm, which is significantly larger than the 6 nm Förster distance of Cy5.^41^ Thus, even if the LNP fusion would lead to complete expulsion of its internal 25% volume of water,^35^ the accompanied reduction in the intermolecular distance between adjacent Cy5 molecules will cause a decrease in the fluorescence emission intensity due to quenching of 5 to 10% at most. We thus exclude photophysical effects as the primary cause of the observed reduction in Cy5-mRNA emission, leaving mRNA release into the solution above the anionic SLB as the most plausible cause for the rapid and dramatic decrease in the Cy5 signal intensity.

The remaining fraction of the Cy5-mRNA intensity (∼30%) continued to reside at or near the LNP docking site after fusion, which can presumably be attributed to entangled mRNA being electrostatically bound to the positively charged headgroup of DLin-MC3-DMA. This molecular complex may, in turn, experience hydrophobic association with lipid assemblies at the site of collapse, which is likely to restrict mRNA translocation across the membrane.

This finding aligns with a recent high-resolution live cell imaging study of mRNA-LNPs of the same type used in this work.^42^ The study showed a gradual transition of mRNA from being confined to the endosomal membrane to a more dispersed state within the maturing endosome, although complete detachment of mRNA from the endosomal membrane was not observed. In contrast, siRNA delivered using the same type of LNPs became homogeneously dispersed in the intraluminal space of the endosome. Consistent with this observation, similar measurements as reported in Figure 2, but for siRNA-containing LNPs, demonstrated complete escape of siRNA into the bulk solution, accompanied by rapid dispersion of Rhod-DOPE into the anionic SLB (Figure S4).

However, it is important to keep in mind that dye-labeled components represent only a small fraction of the molecules participating in the fusion process, which adds uncertainty to any presumptions made about the behavior of the unlabeled components. One way of addressing this concern is to apply dual-color-fluorescence and label-free-scattering microscopy^43,44^ to study the correlation in scattering and fluorescence intensity of the LNPs upon pH changes. Although this method is currently limited to investigations on planar glass substrates, the LNP fusion efficiency with the anionic SLB was observed to be an order of magnitude lower (<15%) than that reported here on porous silica substrates. This difference is attributed to the increased pinning of the SLB to the planar silica compared to the nanoporous silica support, although electrostatically induced alterations in the distribution of POPS between the two bilayer leaflets could also influence the process. However, a similar reduction in Rhod-DOPE emission was observed upon pH-induced fusion of LNPs tethered to the same type of supported anionic SLB (Figure S5). Furthermore, it was evident from changes in the label-free scattering signal that an LNP fusion event is accompanied by a reduction in the scattering intensity by more than 90% (Figure S5). Since the scattering intensity is, to a first approximation, proportional to the square of the LNP mass,^44^ this indicates that a large fraction of the LNP content (∼70% of the mass) dissolves into the anionic SLB and/or escapes into the solution upon fusion.

The analysis depicted so far suggests that at least the majority of the LNP lipid material is efficiently integrated into the underlying anionic SLB during pH-induced LNP fusion. To specifically trace the fate of DLin-MC3-DMA during LNP fusion and disintegration, experiments were conducted using LNPs formed without Rhod-DOPE, but instead with calcein, a highly negatively charged dye^45^ that is expected to be electrostatically bound to the ionized headgroup of the DLin-MC3-DMA lipid upon LNP formation at low pH. Even though the nucleotide (PolyA) encapsulation efficiency was, in this case, reduced by a factor of 3 (Table S1), the release kinetics of calcein observed upon pH-induced LNP fusion (Figure S6) was similar to that observed for Rhod-DOPE, suggesting that a significant fraction of the calcein is transferred into the anionic SLB in the form of calcein-DLin-MC3-DMA complexes, which is further supported by an average diffusion constant of 2.3 μm^2^ s^–1^, being similar to that obtained for Rhod-DOPE (Figure 2g).

### mRNA Remains Bound to the Anionic SLB after LNP Fusion

Considering the molecular transfer from lipid-based drug carriers during interactions with the anionic SLB, it has been previously reported that short siRNAs escape into bulk upon pH-induced electrostatic interactions with charged liposomes designed to mimic the anionic character of the endosomal membrane,^46^ and individual ssDNAs formulated in DOTAP-ssDNA lipoplexes have been shown to remain electrostatically bound to the cationic DOTAP lipids upon electrostatically driven fusion between DOTAP-ssDNA lipoplexes and anionic SLBs.^47^ It is therefore not unlikely that mRNA forms similar complexes with DLin-MC3-DMA lipids, which, if escape into solution occurs, could rebind to the anionic SLB via hydrophobic driven insertion. Alternatively, suspended mRNA could rebind via electrostatic association with ionized DLin-MC3-DMA that escaped into the anionic SLB during LNP fusion. For the latter scenario to be plausible, rebinding is expected to occur near the LNP docking site soon after fusion, before the positive charge of DLin-MC3-DMA is counterbalanced by negatively charged POPS lipids in the SLB. Another plausible explanation for mRNA attachment to the anionic membrane is the formation of lipid-mRNA adducts through covalent addition of reactive lipid species to nucleobases,^48^ known to negatively impact mRNA translation.

**Figure 3:**
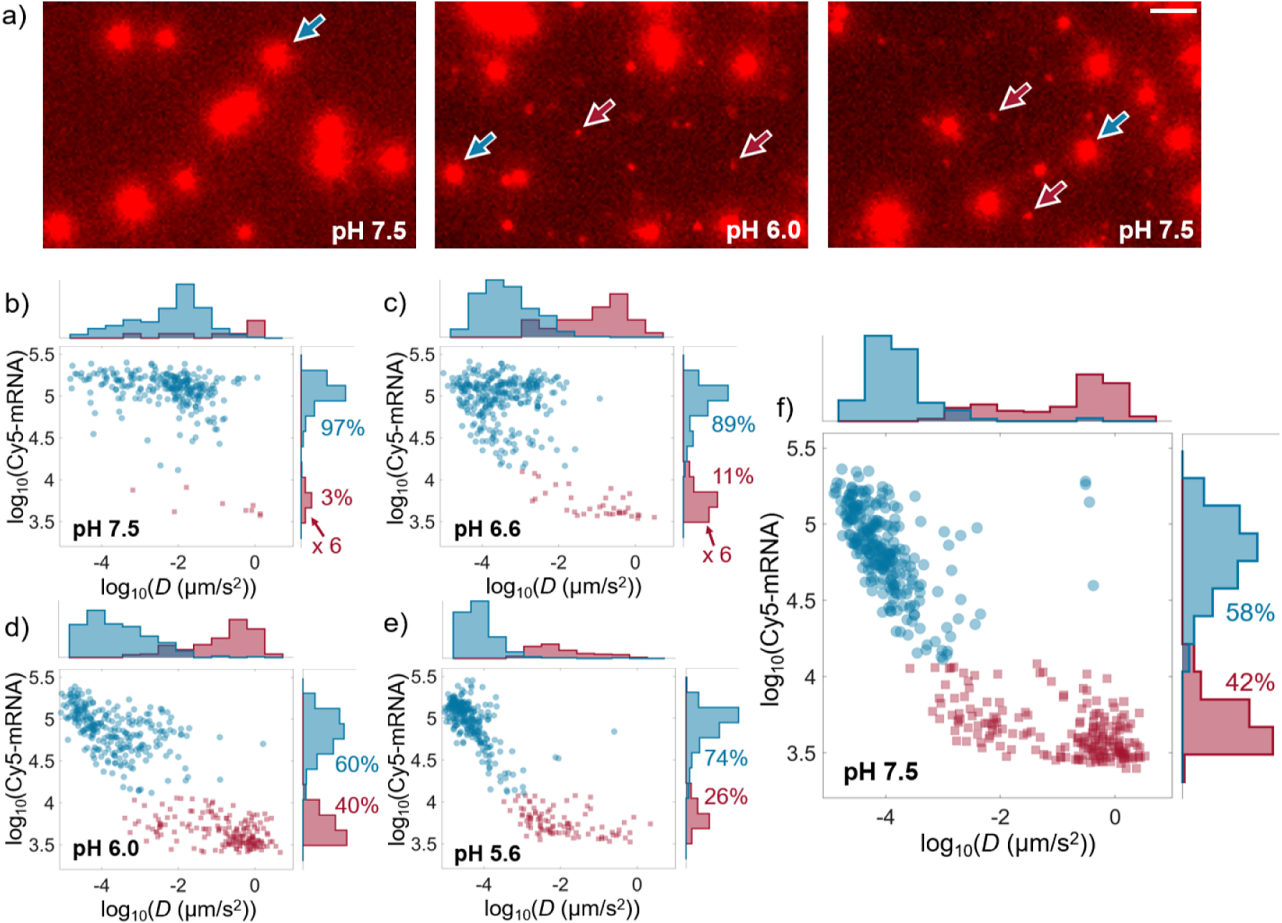
Mobility of membrane-bound mRNA versus pH. a) Cy5-mRNA epi-fluorescence micrographs (from different fields of view) display examples of individual LNPs with high intensity and low mobility (indicated with blue arrows) and low intensity and high mobility (indicated by red arrows) upon tethering at pH 7.5 (left), after reduction to pH 6.0 (middle), and after reverting back to pH 7.5 (right). The scale bar corresponds to 5 μm. b–f) Log–log representation of Cy5-mRNA emission intensity versus diffusion constant *D* for individual detections at pH b) 7.5, c) 6.6, d) 6.0, e) 5.6, and f) after reverting pH back to pH 7.5. To guide the eye, the data is color-coded based on a fluorescence intensity threshold of 4.1. The intensity histogram representing the single mRNA in b and c are multiplied by a factor of 6.

To further elucidate the fate of Cy5-mRNA upon pH-induced fusion, an identical set of experiments was conducted, employing high-intensity epi-illumination at an acquisition rate of 2 fps, facilitating the detection and tracking of weak fluorescence signals (Movie S4). To minimize bleaching effects, the measurements were initiated 5 min after the subsequently induced pH reductions, that is, when essentially no more fusion events were observed (see Figure 1e). These results are summarized, together with representative micrographs (Figure 3a), as scatter plots of the Cy5-mRNA emission intensity plotted versus lateral diffusivity for all individual detectable entities, recorded after the initial LNP binding at pH 7.5 (Figure 3b), and at pH 6.6, 6.0, and 5.6 (Figure 3c–e, respectively), followed by an exchange back to pH 7.5 (Figure 3f).

After initial LNP binding at pH 7.5, the majority of detected entities (∼97%) display a rather uniform intensity distribution with a half-width maximum value corresponding to around 50% of the peak value, which is typically observed for individual LNPs of this type,^49^ but also a small fraction (3%) of entities with around 1.5 times lower intensities when displayed on a log–log scale (Figure 3b). With around 40 Cy5-mRNA molecules per LNP (see above), an individual Cy5-mRNA is expected to have around 1.6 times lower intensity when plotted on a logarithmic scale, suggesting that the low-intensity detections are dominated by individual membrane-bound Cy5-mRNA.

Upon sequential reduction in pH from 7.5 to 5.6 (Figure 3b–e), there is a gradual shift from the high-intensity distribution toward lower intensities, with the most significant change occurring when the pH is lowered from 6.6 to 6.0 (Figure 3d). In this step, there is also a significant increase in the number of individual Cy5-mRNA detections, increasing from 11% to 40% of the detected entities. These observations are attributed to the fact that the fusion efficiency is highest in this step (Figure 1), resulting in a substantial fraction of mRNA being released into solution; however, as evident from this analysis, a small fraction of individual mRNA also rebinds to the anionic SLB. Also, note that there is a reduction in the number of individual Cy5-mRNA detection events at pH 5.6 compared with 6.0 (Figure 3e), tentatively attributed to a reduction in the negative charge of POPS near pH 5.^50^

Since the diffusivity of the anionic SLB may change in response to lipids escaping from fusing LNPs, we refrain from attempting to quantify the diffusivity in terms of the number of contact points between the mobile entities and the SLB. Note, though, that the diffusion constants of individual mRNA range from values similar to those measured for individual lipids (1 to 4 μm^2^ s^–1^) to orders of magnitude lower values, being significative of multiple contact points with the anionic SLB. Additional insights can be gained from inspecting relative changes in the diffusivity distributions when the pH is sequentially reduced from 7.5 to 5.6. First, the diffusivity of the high-intensity distribution shifts toward significantly lower values already when the pH is reduced from 7.5 to 6.6, which is attributed to an increase in electrostatic attraction between partially ionized but nonfused LNPs and the anionic SLB. In contrast, there are no dramatic variations in the diffusivity distribution of individual Cy5-mRNA, except for a reduction in the fraction of mRNA with high diffusivity at pH 5.6, which is attributed to mRNA release due to a reduction in the electrostatic attraction between mRNA and POPS.

These observations might indeed be relevant in the context of endosomal escape since they suggest that mRNA could, in fact, be associated with the endosomal membrane when being translocated to the neutral cytosolic environment. To simulate the endosomal escape step, that is, mRNA translocation from an acidic to a neutral environment, the pH was finally increased from 5.6 to 7.5. If mRNA association is controlled by electrostatic attraction between mRNA and cationic DLin-MC3-DMA present in the membrane, one would expect that deprotonation of DLin-MC3-DMA should be accompanied by the release of Cy5-mRNA into the bulk solution. Instead, the fraction of single Cy5-mRNA increased from 26 to 42%, accompanied by a reduction of the intensity distribution of the high-intensity population (Figure 3f), which is consistent with release of Cy5-mRNA from the site of LNP fusion which, at least in part, remains membrane-bound as Cy5-mRNA monomers. However, although the increase in diffusivity observed for individual Cy5-mRNA in this step indicates a reduction in the number of contact points with the SLB, this experiment cannot conclusively determine whether this phenomenon is attributed to the existence of lipid-mRNA adducts,^48^ or deprotonation-resistant complex salt formation between mRNA and DLin-MC3-DMA, which was recently shown to remain stable in LNPs, even at neutral pH.^51^

To address this question, experiments similar to those presented in Figure 3 were repeated with two differences: (i) POPS was replaced by bis(monoacylglycero)phosphate (BMP), which controls the negative charge in the late endosome and remains negatively charged below pH 4.5, and (ii) the flow rate was adjusted to ensure around 2 orders of magnitude higher shear force acting on membrane-bound molecules. After LNP fusion, induced by reducing the pH to 4.5 to ensure complete ionization of DLin-MC3-DMA, individual Cy5-mRNA displayed low diffusivity at pH 4.5, indicating firm association with the anionic SLB. However, while Cy5-mRNA remained bound at high shear flow at pH 4.5, more than 80% of Cy5-mRNA displayed lateral diffusion upon increasing the pH to 7.5 (Movie S5) followed by detachment (Figure S7), suggesting that protonation-resistant complex salt formation appears to be the dominant reason for the mRNA association at neutral pH at low shear force (Figure 3f), although lipid–mRNA adduct formation may very well explain the fraction of mRNA that still remains bound to the membrane. These results also show that individual membrane-associated mRNA molecules preferentially reside on the upper side of the supported membrane, suggesting inefficient membrane translocation.

### The pH Dependence of LNP Fusion Depends on LNP Formulation

The investigation presented above focuses on an LNP formulation that was previously shown to induce efficient mRNA transfection efficiency.^35^ It was also demonstrated that by approximately doubling the fraction of the gel-phase forming DSPC lipid, known to be predominantly located at the surface of this particular type of the LNP, the protein production was reduced by more than 1 order of magnitude. Since the cellular uptake of these two types of LNPs was observed to not differ significantly, the difference in protein production was attributed to a reduction in endosomal escape efficiency. Since the endosomal escape event is likely to be closely connected with the capacity of LNPs to fuse with the endosomal membrane in response to a reduction in pH, these results motivated us to explore if the minimalistic LNP fusion assay presented in this work could also help elucidate differences in the fusogenicity of these two types of LNPs (see Materials and Methods and Table S1).

**Figure 4:**
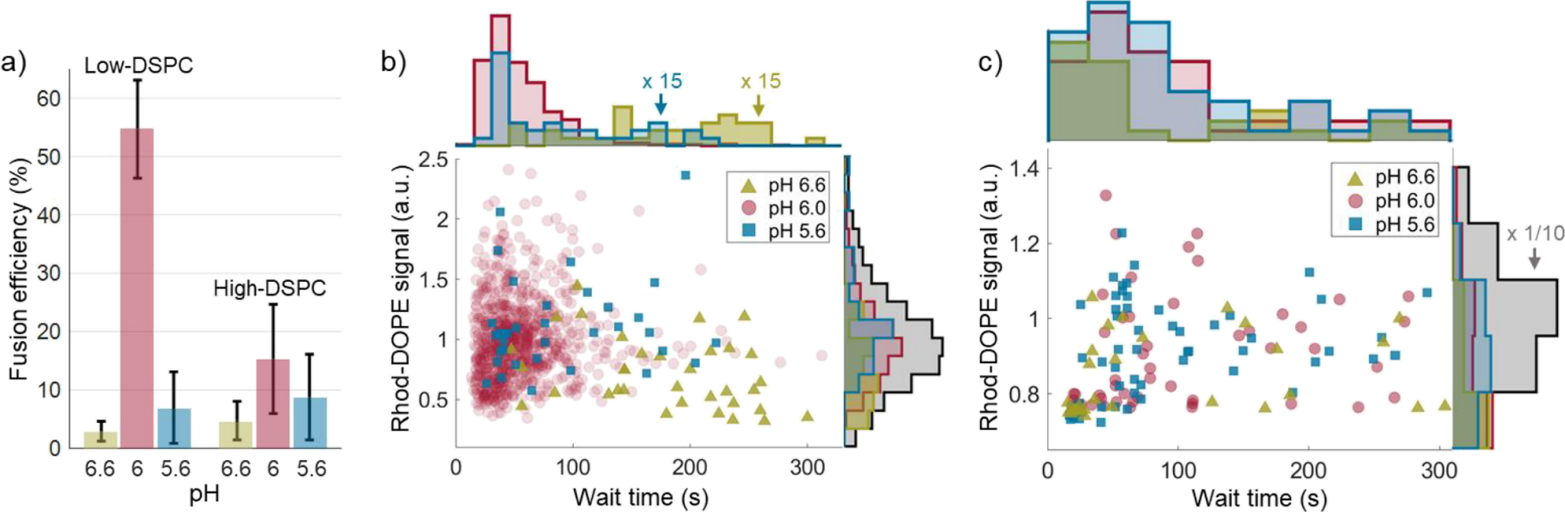
Comparison of the fusion efficiency and characteristics for the two different LNPs. a) Fusion efficiencies at different pH for low-DSPC and high-DSPC LNPs with error bars representing the standard deviation from three measurements. Scatter plots displaying the Rhod-DOPE fluorescence emission intensity at pH 7.5, prior to fusion, versus the wait time to fusion onset together with histograms projected on the respective axis for b) low-DSPC LNPs and c) high-DSPC LNPs. The histograms representing the wait time at pH 5.6 and 6.6 are multiplied by a factor of 15. The gray intensity histogram in c) is divided by a factor of 10. Data were extracted from three independent measurements.

While neither of the two LNP formulations display significant fusion when the pH was dropped from 7.5 to 6.6 (<5%), the LNPs containing a low DSPC concentration (low-DSPC LNPs) displayed at least three times higher (∼55%) fusion efficiency than the high-DSPC LNPs (∼17%) upon a reduction of the pH from 6.6 to 6.0, with cumulative fusion efficiencies of ∼62 and 21%, respectively, upon subsequent reduction of the pH to 5.6 (Figure 4a). It is also worth noting that low- and high-DSPC LNPs contain 0.7 and 0.25% DMPE-PEG2000, respectively, which converts to distances between surface-associated PEG chains of approximately 2.5 and 4.1 nm, respectively. With a Flory radius of ∼3.5 nm for 2 kDa PEG,^52^ this suggests a PEG brush conformation for low-DSPC LNPs, which should intuitively prevent close contact with the anionic SLB to a greater degree than the lower PEG formulation. However, under the reasonable assumption that DMPE-PEG2000 remains bound to the LNPs after surface attachment, the results show that potential steric repulsion induced by the presence of PEG seems to be overcome by the pH-induced electrostatic attraction, and that the high surface concentration of the gel-phase forming DSPC lipid is the dominating reason for the lower fusion efficiency of high-DSPC LNPs. This observation also supports that the previously reported^35^ difference in protein production for these two LNPs is indeed most likely due to a difference in endosomal escape efficiency caused by the difference in the surface concentration of DSPC.

Furthermore, by exploring differences in the fusion behavior at different pH, additional mechanistic insights can be gained. Figure 4b,d summarize the key results of this set of experiments in scatter plots displaying the Rhod-DOPE fluorescence emission, which, to a good approximation, represents a measure of LNP size,^49^ prior to fusion at pH 7.5 versus the wait time (Figure 1e) between the rapid pH drop (<1 s) and the onset of fusion, together with histograms projected toward the respective axis. Focusing on the low-DSPC LNPs first (Figure 4b), the average wait time is on the order of 200 s upon reducing the pH from 7.5 to 6.6, which is around 10 times longer than the corresponding average wait time for the fusion events observed upon subsequent reduction of the pH from 6.6 to 6.0 (around 20 s). It is also clear that upon reducing the pH from 7.5. to 6.6, it is preferentially small (low Rhod-DOPE emission) LNPs that undergo fusion, while at pH 6.0, the LNPs that undergo fusion display a size distribution similar to the original distribution at pH 7.5. Conversely, for high-DSPC LNPs, the fusion efficiency is markedly lower and the statistics is therefore low. Nonetheless, it is evident that there is a broad distribution of wait times at all pH levels, with a tendency toward increased fusion efficiency and shorter wait times for small (low Rhod-DOPE emission) LNPs (Figure 4c).

Despite these differences between low- and high-DSPC LNPs, the TNS assay shows essentially identical transitions around an inflection point at pH 6.6 for both types of LNPs (Figure S2). Under the assumption that DLin-MC3-DMA is homogeneously distributed within the LNP at neutral pH and that the fraction of ionized DLin-MC3-DMA is independent of LNP size, the surface coverage of ionized DLin-MC3-DMA that can potentially be reached should scale as the inverse of the LNP radius. One plausible explanation for the observation showing that small LNPs tend to fuse more efficiently at pH 6.6 (Figure 4b,c), at which only a fraction of DLin-MC3-DMA is ionized, is therefore that only LNPs in the smaller size regime gain sufficient surface charge for the electrostatic attraction to become high enough for fusion to occur. However, if one assumes that up to one-third of DLin-MC3-DMA can be engaged in a complex salt with mRNA,^51^ there are on the order of 450 × 10^3^ DLin-MC3-DMA available for ionization within a 140 nm diameter LNP, while the maximum number of lipids (assuming a headgroup area of ∼1 nm^2^) located at the LNP interface is around 60 × 10^3^. Thus, if 50% of DLin-MC3-DMA is ionized already at pH 6.6, the amount of ionized DLin-MC3-DMA is unlikely to be the limiting factor for fusion. Rather, the fusion event seems to be limited by the capacity of DLin-MC3-DMA to be sufficiently and favorably exposed at the LNP interface, which is likely to include a process in which DSPC, cholesterol, and PEGylated lipids are replaced or expelled. Such mechanisms may very well vary across the LNP size and also be influenced by size-dependent variations in the surface concentration of gel-phase forming DSPC as well as differences in the interfacial membrane strain.

It is also worth noting that although the fusion efficiency of low-DSPC LNPs is as high as 60%, there is still a significant fraction of the tethered LNPs that do not undergo fusion. Together with the variations in wait time and loss of mRNA observed between individual low-DSPC LNPs, this suggests that not only are there significant differences between the two types of LNPs investigated in this work but also significant heterogeneity within an individual LNP population. This is indeed evident also for the small fraction of high-DSPC LNPs that undergo fusion, which display significant variations with respect to the wait-time prior to fusion; yet, irrespective of LNP type and size, essentially no fusion events were observed with wait times shorter than around 5 to 10 s even at pH 6.0 and 5.6 (Figure 4b,c), suggesting that also for LNPs with the “optimal” structure, it takes several seconds for ionized DLin-MC3-DMA to be favorably exposed on the LNP interface for fusion to occur.

## Conclusions

We acknowledge that the minimalistic approach used to mimic the early endosomal membrane excludes the complexity of natural endosomal membranes, which contain a diverse lipid composition and various types of membrane proteins.^53^ However, this simplification isolates the impact of electrostatics, which is crucial in the context of pH-induced fusion with the early endosome. Thus, considering the importance of the pH-induced onset of electrostatically driven LNP association and disintegration at the endosomal membrane, the fundamental mechanisms and distinct features observed in this work are likely to resemble those occurring in a natural endosomal environment, in particular, the likely presence of mRNA escape into the solution followed by rebinding of monomeric mRNA to the ionizable-lipid-containing endosomal membrane, as well as the presence of multiple entangled mRNA molecules that remain associated with the site of fusion (Figure 3). This suggests two plausible mRNA escape mechanisms: (i) disentanglement from mRNA at the site of fusion into the neutral cytosolic environment, or (ii) escape of individual mRNA bound to the endosomal membrane through pores in the membrane caused by endosomal remodeling and disintegration by transfer of ionized lipids from the LNP.

In this context, it is worth noting that in our experiments, it seems that only a minor fraction of mRNA that escapes into the solution upon LNP fusion (Figure 3) rebinds to the anionic SLB. However, given the low surface-to-liquid volume ratio in the microfluidic channel, this is expected. If this process occurs for LNPs bound to the membrane of a closed endosomal compartment with a submicrometer dimension, all suspended mRNA molecules would reside in close proximity to the inner endosomal membrane. Such conditions would elicit a high probability of mRNA-membrane binding, mediated through, for example, ionized lipids that escape into the membrane during LNP fusion or because suspended mRNA is complexed with ionized lipids. Considering the presence of this phenomenon, it is not obvious whether mRNA translocation into the neutral cytosolic environment, which is expected to be a very rare process, is more likely to occur through translocation of individual endosomal-membrane-bound mRNA in response to endosomal damage,^17,23^ or whether mRNA manages to escape prior to endosomal damage from the entangled mRNA state that we observed remaining at the site of fusion (Figure 2). Furthermore, the fact that the complex salt formed between mRNA and DLin-MC3-DMA under acidic conditions is not instantaneously reversed at neutral pH^51^ is yet another factor that may contribute to the low transfection efficiency, since it may hamper the efficiency of ribosomal protein synthesis or lead to undesired mRNA association with internal cellular membrane compartments.

It is also worth noting that although the simplistic model of the early endosomal membrane helps identify general features in the temporal dynamics of pH-induced LNP fusion and subsequent disintegration, inspection with a single LNP resolution helped identify significant differences between different types of LNPs as well as heterogeneity among individual LNPs of the same type. For example, there is a wide distribution in the wait time between a reduction in pH and the onset of LNP fusion, lasting between tens and hundreds of seconds depending on the pH, size, and type of LNPs. In particular, the LNP size was observed to influence the wait time prior to fusion at a moderate reduction in pH (Figure 4). Although the stoichiometry of the LNP components may still vary with size within a single batch, the variation in composition is not expected to be as significant as when the LNP size is adjusted by altering the relative amounts of the LNP components, thus highlighting the value of single LNP resolution in the context of identifying the relative importance of different LNP properties. Furthermore, around 40% of the most fusogenic type of LNP (low-DSPC LNPs) did not undergo fusion even when the pH was eventually reduced to 5.6 (Figures 1 and 4), at which the majority of the ionizable lipids are expected to be ionized. Thus, even if ionizable lipid-containing LNPs serve to provide very efficient mRNA encapsulation, typically exceeding 95%, and the correct choice of helper lipids can provide sufficient stability and circulation times for efficient cellular uptake, we dare to conclude from this study that there are significant variations between individual LNPs with respect to their fusion capacity. For example, the large size of the mRNA cargo compared with the lipid components of an LNP suggests that the LNP structure could be quite sensitive to variations in mRNA content, particularly in the small size regime. Consequently, the fates of individual mRNA molecules encapsulated in the same LNP may also differ. This, in turn, calls for efforts devoted to refined LNP fabrication protocols with the capacity to produce LNP batches with significantly reduced variations. Furthermore, in agreement with cellular data, almost 1 order of magnitude more efficient fusion was observed for LNPs with reduced surface density of gel-phase forming DSPC lipids, despite that both low- and high-density DSPC LNPs display identical charge titration curves using the TNS assay.

In summary, assays like the one reported in this work, which extend beyond the characterization of the LNP size, encapsulation efficiency, structure, and surface charge, can serve as valuable tools for understanding the physicochemical mechanisms underlying pH-induced LNP fusion with anionic membranes and may also help identify formulations with preferred features and the desired functional response. Given the simplicity of the early endosomal membrane mimic used to isolate electrostatic effects, it is crucial to emphasize that the heterogeneity in LNP fusion is likely to be even more significant in the natural cellular environment. This is especially true since LNPs acquire a protein corona prior to endocytic uptake,^54^ a process known to influence their pH-dependent interaction with anionic membranes similar to the one used in this work.^27^ Future work should, therefore, focus on designing more realistic mimics of the dynamically varying endosomal membrane, preferably also representing the curved shape of the endosome, with an emphasis on LNPs that have more subtle compositional differences than the ones used here but still display significant differences in cellular and in vivo efficacy. The use of specific labeling of the ionizable lipid,^42^ as well as the different helper lipids, could provide additional information regarding the spatiotemporal evolution upon pH-induced LNP fusion, while neutron reflectometry combined with selective deuteration procedures could provide quantitative estimates of lipid transfer.

## Materials and Methods

### LNP Composition

The low- and high-DSPC LNP formulations contained ionizable cationic lipid *O*-(*Z*,*Z*,*Z*,*Z*-heptatriaconta-6,9,26,29-tetraem-19-yl)-4-(*N*,*N*-dimethylamino)butanoate (DLin-MC3-DMA), 1,2-distearoyl-*sn*-glycero-3-phosphocholine (DSPC), cholesterol, 1,2-dimyristoyl-*sn*-glycero-3-phosphoethanolamine-*N*-[methoxy(polyethylene glycol)-2000] (DMPE-PEG2000), 1,2-distearoyl-*sn*-glycero-3-phosphoethanolamine-*N*-[biotinyl(polyethylene glycol)-2000] (DSPE-PEG2000 Biotin) and 1,2-dioleoyl-*sn*-glycero-3-phosphoethanolamine-*N*-(lissamine rhodamine B sulfonyl) (Rhod-DOPE) in molar ratios given in Table S1. The LNP cargo contained Cy5-labeled (TriLink BioTechnologies) and nonlabeled eGFP-encoding mRNA at a 1:4 molar ratio. According to the manufacturer, the ratio of Cy5-labeled and unlabeled uridine (U) is 1:3. The open reading frame of eGFP contains 103 U, which gives 26 Cy5-U. Additionally, there are another 277 nucleotides in the full sequence with ∼120–150 estimated to make up the poly(A)-tail.^55^ If one assumes that 25% of the remaining 120–160 nucleotides are U, we expect an additional 7–9 Cy5-U, resulting in a total of ∼34 Cy5-U per mRNA. The PolyA- and calcein-containing LNPs were prepared from an aqueous solution containing 30 mM calcein and eGFP-mRNA replaced with PolyA (Table S1).

### LNP Preparation and Characterization

Low- and high-DSPC LNPs were prepared using the NanoAssemblr Benchtop device, while siRNA and PolyA- and calcein-containing LNPs were prepared using the NanoAssemblr Spark device (both from Precision Nanosystems Inc., Canada). Briefly, stocks of lipids were dissolved in ethanol and mixed in appropriate molar ratios to obtain a lipid concentration of 12.5 mM. mRNA or PolyA were diluted in RNase-free citrate buffer (Teknova) of 50 mM at pH 3.0 to obtain an mRNA/PolyA:lipid weight ratio of 1:10. The aqueous and ethanol solutions were mixed in a 3:1 volume ratio through a microfluidic cartridge of the Benchtop device at a flow rate of 12 mL min^–1^. LNPs were dialyzed overnight against 600× sample volume nuclease-free PBS using Slide-A-Lyzer G2 dialysis cassettes (Thermo Scientific) with a molecular weight cutoff of 10 K. The collected LNPs with an mRNA concentration of about 0.1 mg/mL were filtered through a sterile filter (0.2 μm) prior to use. The size of the LNPs was determined by DLS measurements using a ZetaSizer Nano ZS from Malvern Instruments Ltd. The encapsulation efficiency was determined using the RiboGreen assay (ThermoFisher). The LNP size and concentration were also determined using nanoparticle tracking analysis (NTA) using a Nanosight LM10 device with a Hamamatsu C11440–50B/A11893–02 camera. The anionic fluorescent dye 2-(*p*-toluidino)-6-naphthalene sulfonic acid (TNS) measurements were performed in a 384-well format with a buffer containing 20 mM phosphate tribasic, 25 mM ammonium citrate, 20 mM ammonium acetate, and 150 mM sodium chloride, with a pH ranging from 2 to 11. The molar ratio of total lipid:TNS dye was maintained at 4.25, and the total lipid concentration in each well was maintained at 7.3 μM. All measurements were performed at room temperature within 10 min of preparation using a fluorescence plate reader (BMG Labtech) with excitation at 340 nm and emission at 460 nm.

### Composition of the Anionic SLB

Lipids used to produce the lipid vesicles for SLB formation were purchased from Avanti Polar Lipids, Inc. The lipid vesicles were produced by the lipid film hydration and extrusion method. In brief, 1-palmitoyl-2-oleoyl-*sn*-glycero-3-phosphocholine (POPC), 1-palmitoyl-2-oleoyl-*sn*-glycero-3-phospho-l-serine (POPS), 1,2-dioleoyl-*sn*-glycero-3-phosphoethanolamine-*N*-(cap biotinyl) (Biotin-Cap-DOPE), and 1,2-dioleoyl-*sn*-glycero-3-phosphoethanolamine-*N*-(7-nitro-2–1,3-benzoxadiazol-4-yl) (NBD-DOPE) were suspended in chloroform at concentrations of 10, 10, 0.5, and 1 mg mL^–1^, respectively. 186.30 μL of POPC (93.45 mol %), 12.34 μL of POPS (6 mol %), 2.9 μL of Biotin-Cap-DOPE (0.05 mol %), and 12.12 μL of NBD-DOPE (0.5 mol %) were mixed and dried in a vacuum overnight. The lipid film was rehydrated with PBS for 1 h to a total lipid concentration of 2 mg mL^–1^. The solution was subsequently extruded 21 times using a mini extruder (Avanti Lipids Inc., Alabaster, AL, USA) with 50 and 30 nm polycarbonate membranes (Whatman, Maidstone, UK) to form vesicles with the diameter of approximately 100 nm. The vesicle solution was stored at 4 °C for later use. The zeta potential ζ for the lipid vesicles was determined using ZetaSizer (Malvern) to be −22.6 ± 2.05 V.

### Nanoporous Silica Thin Film Formation

Nanoporous silica thin films were synthesized by following a modified method by Alberius et al.^56^ Briefly, 0.28 g of poly(ethylene glycol)-*block*-poly(propylene glycol)-*block*-poly(ethylene glycol) (P123, Sigma-Aldrich) was dissolved in 1.33 g of ethanol (99.5%, Solveco) in a glass vial. This mixture was stirred using a magnetic stirrer at room temperature until completely dissolved. In a separate vial, 1.73 g of tetraethylorthosilicate (TEOS, 98%, Sigma-Aldrich) and 2 g of ethanol were combined and stirred at 300 rpm with a magnetic stirrer. Subsequently, 0.9 g of 0.01 M HCl (Sigma-Aldrich) was added dropwise to this mixture and stirred continuously for 20 min. After this period, the P123 solution was mixed with the TEOS solution. This silica precursor solution was then stirred at room temperature at 300 rpm for 20 min to achieve a homogeneous and clear solution.

The silica precursor solution was deposited onto borosilicate cover glasses (Menzel-Gläser, D263, number 1) through spin-coating at 4000 rpm (WS-650, Laurell Technologies Corporation). This was done immediately after submerging the glasses in an EtOH–NaOH (5:1) cleaning solution for 5 min, followed by a thorough rinse with ultrapure water (Milli-Q, Merck Millipore), and drying using nitrogen gas. The coated glasses were then left in the dark to age at room temperature for 24 h. The templating agent was removed by gradual heating at a rate of 1 °C per minute from room temperature to 400 °C and maintaining this temperature for 4 h before allowing cooling to room temperature. Top-view SEM (scanning electron microscopy) analysis of the silica thin films formed on silicon wafers was performed using a Leo Ultra 55 FEG SEM (Zeiss) at an operating voltage of 1 kV.

### Formation of the Anionic SLB

To prepare the microfluidic channel, the Ibidi sticky microfluidic channels (3.8 × 17 × 0.4 mm in width × length × height, Ibidi cell in focus, Gräfelfing, Germany) were attached to the nanoporous substrate after first being cleaned thoroughly using ethanol and water followed by two subsequent steps of UV ozone treatment (for ∼20 min), Milli-Q/ethanol rinsing, and nitrogen drying. Anionic SLBs were formed on the porous substrate by injecting the lipid vesicle suspension (diluted to a lipid concentration of 200 μg mL^–1^) into PBS. NeutrAvidin suspended in PBS (∼20 μg mL^–1^) was injected in the channel at a flow rate of 50 μL per minute for 10 min followed by thorough rinsing for 5 min with citrate-phosphate buffer (pH 7.5). The LNP stock solution was diluted 1000 times in phosphate-citrate buffer (pH 7.5) and injected at a flow rate of 140 μL min^–1^ until a suitable LNP coverage was obtained (see main text), followed by 5 min of rinsing in pure buffer. To vary the pH from 7.5 to 5.6, we used a citrate-phosphate buffered saline with 150 mM NaCl.

### Fluorescence Microscopy

For time-resolved imaging, the microfluidic system was mounted on an inverted Eclipse Ti-E microscope (Nikon Corporation, Minato City, Japan) equipped with a CFI Apo TIRF 60× (NA 1.49) oil immersion objective (Nikon Corporation, Tokyo, Japan). A FITC filter set (Semrock, Sandwich, IL, USA) was used to excite the NBD-DOPE dyes for visualizing lipid vesicle adsorption and SLB formation on the nanoporous silica substrate. In addition, the continuity and fluidity of the bilayer were evaluated using FRAP assessment by bleaching NBD lipids in a circular region (spot) of the SLB with a solid-state light source (Lumencor Spectra X-LED) at a wavelength of 531 nm, followed by imaging of the fluorescence recovery at 12 fps. FRAP data were analyzed using a custom-written code^36^ in MATLAB (MathWorks, Inc., USA). LNP binding to the anionic SLB was observed using TIRF microscopy, utilizing a TRITC filter set (Semrock, Sandwich, IL, USA) for the Rhod-DOPE dyes (excitation: 565 nm; emission: 590 nm) and a Cy5 ET filter set (F46–006 ET-set, Chroma Technology Corporation, USA) for the Cy5 dyes (excitation: 640 nm; emission: 670 nm), conjugated to the mRNA cargo. Imaging was performed for Rhod-DOPE and Cy5-mRNA separately, at an exposure time of 50 ms with a frame rate of 3 and 2 fps, respectively. Simultaneous imaging of Rhod-DOPE and Cy5-mRNA was performed using an image splitter (OptoSplit II, Cairn Research) at 20 fps.

### Image Analysis

The positions of individual signals in the fluorescence micrographs were determined using threshold-based maxima detection, followed by a subpixel position determination employing radial symmetry characteristics.^57^ For time-resolved videos, where intensity extraction for numerous signals was done (as shown in Figures 1, 3, and 4), the signal positions were linked into trajectories using the Hungarian algorithm.^58^ The emission intensities were extracted from the background-subtracted micrographs as the sum of pixel values in a quadratic area with the center defined by the position and the side length selected to reflect the average extension of signals in the micrographs.

Fusion events were detected automatically using a custom-written script, with a fusion event defined as a relative intensity change of >30% within <5 consecutive frames, generally also coinciding with the loss of the particle trajectory. For data displaying particularly low fusion efficiency, e.g., at a pH decrease from 7.5 to 6.6, fusion events were marked manually using the Point Picker plugin (Philippe Thévenaz, Biomedical Imaging Group, Swiss Federal Institute of Technology, Lausanne) of ImageJ and later matched with specific particle trajectories.

The temporal evolution of intensity profiles used for the extraction of lateral Rhod-DOPE diffusivity *D* was analyzed by a two-dimensional Gaussian function fitted to a 34 × 34 pixels centered around the intensity profile maximum. The emission intensity of the particle and the region surrounding the particle were extracted as the sum of values of the Gaussian fit in an area defined by a maximum distance of 4 pixels and a distance between 4 and 16 pixels to the signal center, respectively. The emission intensity of the particle and the surrounding area both represent background-subtracted intensities, as the Gaussian function was shifted to a zero offset prior to a fusion event. The relative change of emission intensity upon fusion was determined from the change in total emission intensity obtained from the integral of the Gaussian fit. The lateral particle diffusion was determined as previously described^59^ by extracting the orthogonal position variations Δ*x* and Δ*y*, subsequently used to estimate the mean square displacement MSD_i_ = < Δ_i_^2^ >, from which the 1D diffusion coefficient was obtained as *D*_i_ = MSD_i_/(4 Δ*t*), for *i* = *x*, *y*. Finally, the 2D diffusion coefficient *D* was calculated as the arithmetic average of *D*_*x*_ and *D*_*y*_. All data analysis was performed using MATLAB (MathWorks, Inc., USA).

## Supporting Information Available

Structures of the lipids used (Figure S1); the TNS fluorescence assay (Figure S2); time-resolved variation in Cy5-mRNA emission intensity measured by epi-mode microscopy (Figure S3); pH-induced fusion of Atto647-siRNA containing LNPs (Figure S4); label-free scattering microscopy measurements of pH-induced fusion of low-DSPC LNPs (Figure S5); pH-induced fusion of calcein-containing LNPs (Figure S6); pH-induced fusion for low-DSPC LNPs on a BMP-containing SLB (Figure S7); lipid compositions and characteristics of LNP formulations (Table S1) (PDF)
LNP fluorescence emission from pH 7.5 to 6.5 (Movie S1) (AVI)
LNP fluorescence emission at pH 6 (Movie S2) (AVI)
LNP fluorescence emission at pH 5.6 (Movie S3) (AVI)
High-intensity epi-illumination of Cy5-mRNA (Movie S4) (AVI)
Comparison of Cy5-mRNA bound at high shear flow at pH 4.5 with Cy5-mRNA displaying lateral diffusion increasing the pH to 7.5 (Movie S5) (AVI)

## Supplementary Material

nn4c04519_si_001.pdf

nn4c04519_si_002.avi

nn4c04519_si_003.avi

nn4c04519_si_004.avi

nn4c04519_si_005.avi

nn4c04519_si_006.avi
