# Supplementary material for: Time-Resolved Inspection of Ionizable Lipid-Facilitated Lipid Nanoparticle Disintegration and Cargo Release at an Early Endosomal Membrane Mimic
Source: ACS Nano. 2024 Aug 12;18(34):22989–3000. doi: 10.1021/acsnano.4c04519 (PMC11363135; doi:10.1021/acsnano.4c04519)
Supplement: Supplementary file 1 — nn4c04519_si_001.pdf [file nn4c04519_si_001.pdf]

Supporting Information

Time-Resolved Inspection of Ionizable Lipid-Facilitated Lipid Nanoparticle Disintegration and Cargo Release at an Early Endosomal Membrane Mimic

Nima Aliakbarinodehi[1*], Simon Niederkofler[1*], Gustav Emilsson[2], Petteri Parkkila[1], Erik Olsén[1], Yujia Jing[2], Mattias Sjöberg[1], Björn Agnarsson[1], Lennart Lindfors[2], Fredrik Höök[1#]

1) Chalmers University of Technology, Department of Physics, division of Nano and Bio Physics, Fysikgränd 3, 41296 Göteborg, Sweden

2) Advanced Drug Delivery, Pharmaceutical Sciences, R&D, AstraZeneca, 43181 Mölndal, Sweden

#) Corresponding author: fredrik.hook@chalmers.se

SUPPLEMENTARY FIGURES

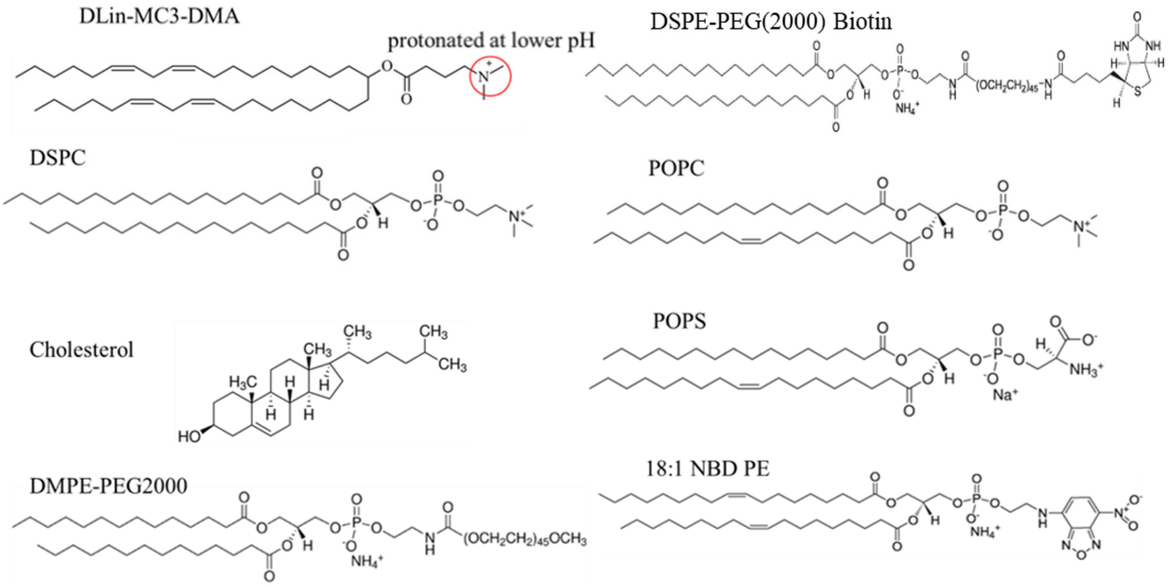

Figure S1. Structures of lipids used in lipid nanoparticle (LNP) and supported lipid bilayer (SLB) preparations.

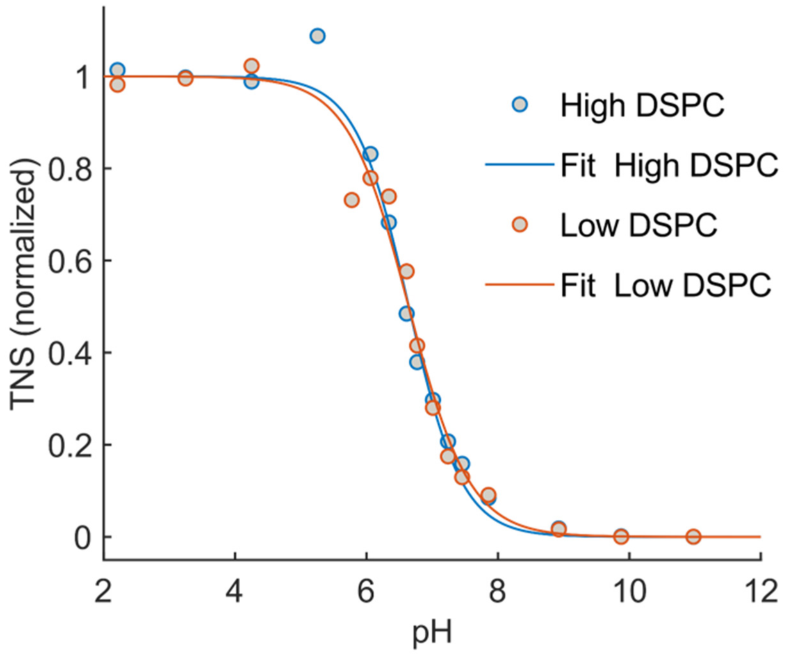

Figure S2. In situ TNS fluorescence titration of low- (blue circles) and high-DSPC (red circles) LNPs. Duplicate measurements were averaged and fitted (solid lines) to a three-parameter sigmoidal.

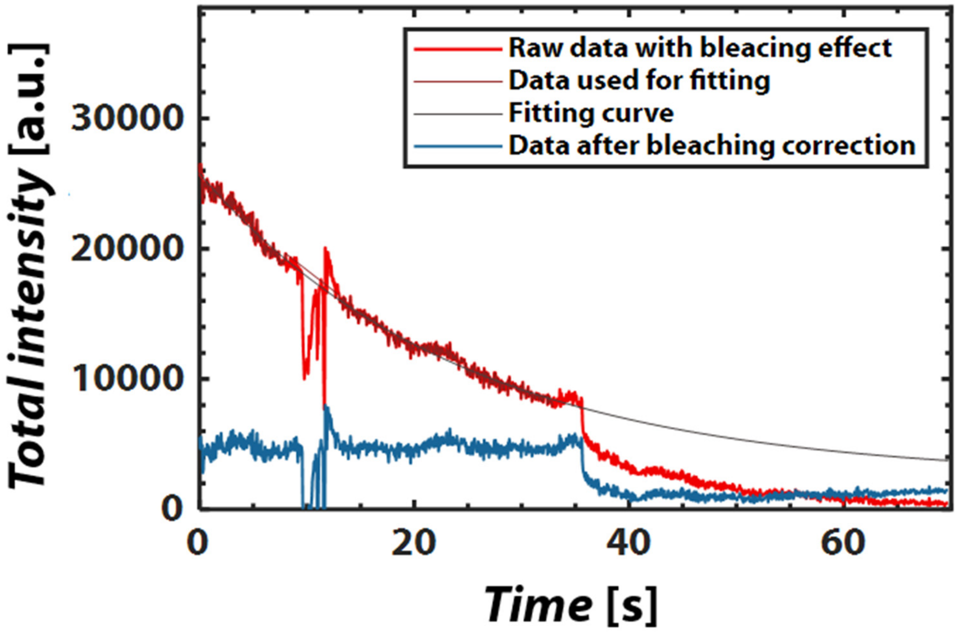

Figure S3. Representative example of time-resolved total Cy5-mRNA emission intensity changes upon reduction of the pH from 6.0 to 5.6 extracted from a single LNP visualized in epi-fluorescence mode. The red curve represents raw data, and the blue curve represents the bleaching-corrected data.

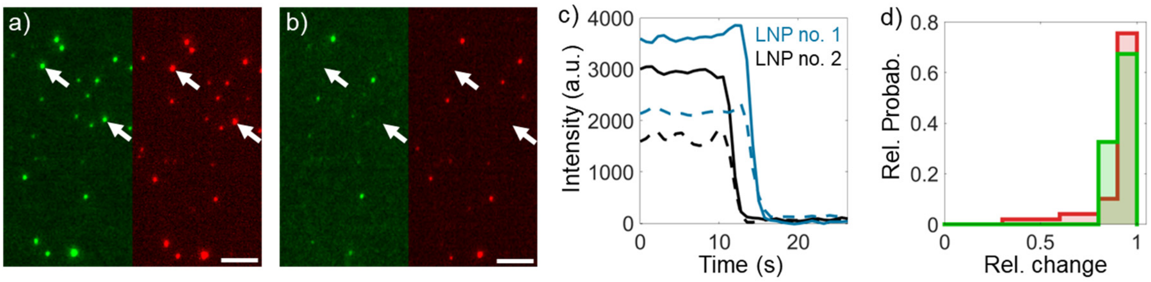

Figure S4. Time-resolved inspection of pH induced siRNA-LNP fusion and cargo escape. TIRF micrographs showing emission signals for (left) Rhod-DOPE, and (right) Alexa Fluor 647 (AF647) labeled siRNA (AllStars Negative Control siRNA, Qiagen) for LNPs (50 mol% DLin-MC3-DMA, 10 mol% DSPC, 38.44 mol% Chol, 1.494 mol% DMPE-PEG(2000), 0.006 mol% DSPE-PEG(2000) Biotin, 0.06 mol% Rhod-DOPE) tethered to a nanoporous supported anionic lipid bilayer (see main text) measured at 20 fps upon a reduction in pH from **a)** 7.5 to **b)** 5.6 (scale bar 5 µm). **c)** Background-subtracted spatiotemporal emission profiles for individual LNPs were fitted to two-dimensional Gaussian profiles that are represented as time-resolved one-dimensional averages for Rhod-DOPE (solid lines) and AF647-siRNA (dashed lines), respectively. **d)** Relative change $\left(\frac{I_f(t=0)-I_f(t)}{I_f(t=0)}\right)$ in the Rhod-DOPE (green) and AF647-siRNA (red) emission intensities for LNPs that undergo pH-induced fusion. The data are consistent with essentially complete dispersion of Rhod-DOPE into the anionic SLB and complete escape of siRNA into bulk.

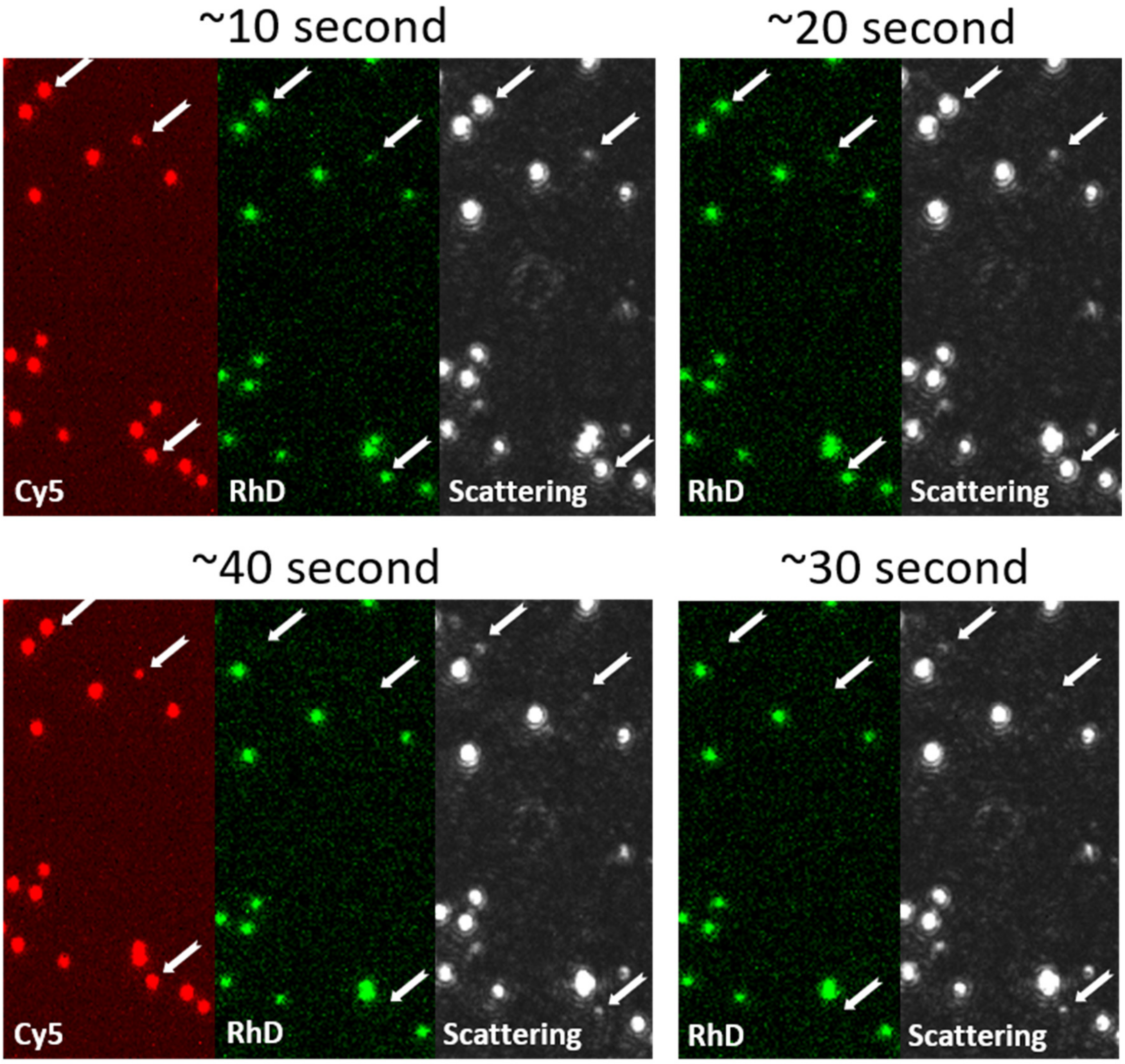

Figure S5. Time resolved dual-mode fluorescence and label-free-scattering micrographs measured upon a reduction of the pH from 6.0 to 5.6 for low-DSPC LNPs tethered via NeutraAvidin to the same type anionic SLB as used in the main text, but here formed on a planar glass of a Nanolyze Sense waveguide chip (Nanolyze AB)[1]. An Olympus BX61 microscope was used for image acquisition (objective: 60X, NA 1.0, camera: ORCA-Flash 4.0 V2.0 CMOS, image splitter: Hamamatsu W-VIEW GEMINI, lasers: Cobolt 06-MLD, λ = 488 nm, and OZ 3000, λ = 635 nm). Both the Rhod-DOPE and the scattering signal display a significant decrease at ~30s, consistent with fusion, while the reduction in the Cy5-mRNA signal is less dramatic. The magnitude of the decrease in the scattering signal is >90%.

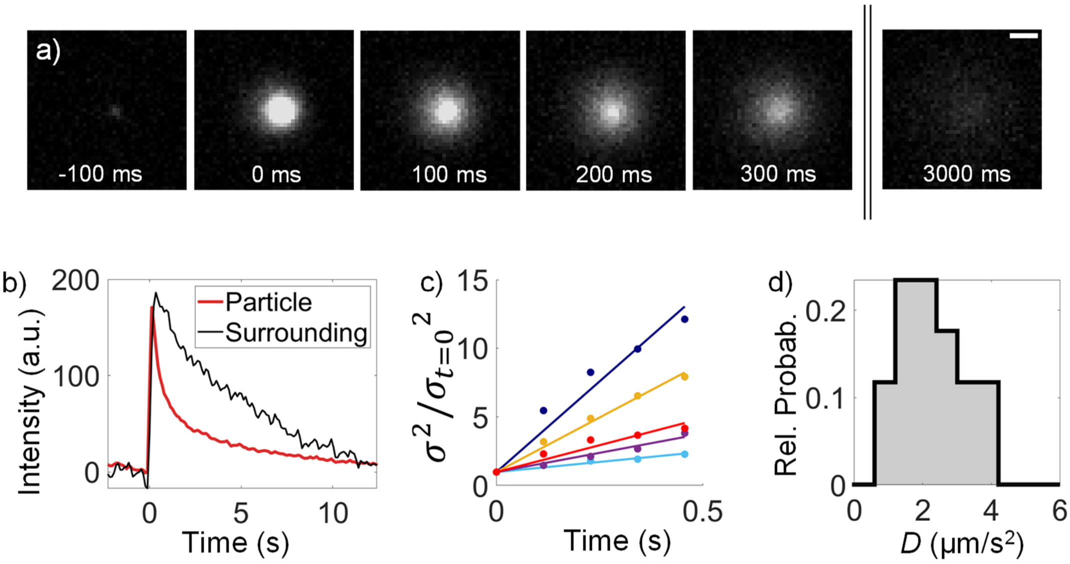

Figure S6. TIRF micrographs of a tethered calcein-containing LNPs, c.f. Fig. 2 in the main text (scalebar 1 μm). **b)** The time-evolution of the total intensity emission (red lines) and the emission from an area surrounding the LNP docking site (black lines) extracted from background subtracted emission profiles represented by two-dimensional Gaussian profiles (Fig. 2 in main text). The initial increase in the total intensity is attributed to calcein dequenching upon LNP collapse, followed by lateral escape of calcein-DLin-MC3-DMA complexes in the endosomal membrane mimic. The higher total intensity in the area surrounding the LNP is attributed to a combination of a temporal increase due to dequenching and higher illumination intensity experienced by the fluorophore as it moves closer to the glass interface upon LNP collapse. **c)** $\sigma^2/\sigma_{t=0}^2$ versus time, with the variance obtained from the Gaussian representation of the LNPs **d)** Distribution of diffusion constants, *D,* obtained from 17 different LNP-variances, $\sigma^2 = 2Dt$, from c).

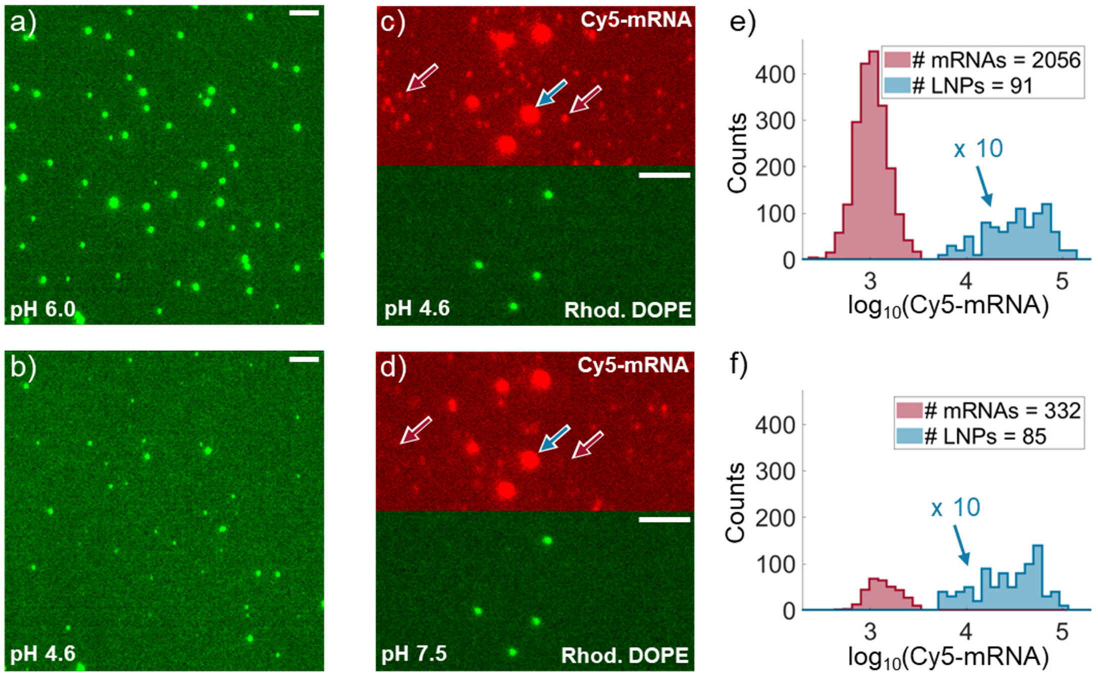

Figure S7. Biotin-modified low-DSPC LNPs were bound to a NeutrAvidin-modified SLB formed on nanoporous silica. The SLB contained POPC, bis(monoacylglycero)phosphate (BMP), DOPE-NBD, and DOPE-Cap-Biotin in a molar ratio of 89.7:10:0.25:0.05. LNP binding was achieved through solution exchange in a flow cell (1.0×17×0.1 mm in width×length×height) at a volumetric flow rate of 5 µL min^{-1} until an LNP coverage of ~0.02 mm^{-2} was reached. LNP binding was terminated by rinsing the channel with pH 7.5 buffer solution at a volumetric flow rate of 200 µL min^{-1}. This was followed by pH reduction via rapid liquid exchange with **a)** pH 6.0 and **b)** pH 4.6 buffers at a volumetric flow rate of 50 µL min^{-1}, leading to fusion of ~5% and ~60% of LNPs, respectively. This was followed by continuous epi-fluorescence imaging at a volumetric flow rate of 400 µL min^{-1}, at **c)** pH 4.6 followed by **d)** an increase in the pH to pH 7.5. The mRNA-Cy5 and Rhod-DOPE emission revealed unfused LNPs, characterized by Cy5-mRNA and Rhod-DOPE emission signals like those of LNPs after binding to the SLB (blue arrow in micrographs c and d). Entities displaying no Rhod-DOPE emission and a significantly lower Cy5-mRNA emission than that of LNPs (red arrows in micrographs) are attributed to individual membrane-bound Cy5-mRNA (cf. Fig. 3, main text). Comparison of unfused LNPs and single mRNA, distinguished by threshold-based clustering of the Cy5-mRNA emission, at **e)** pH 4.6 and **f)** after an increase to pH 7.5, reveals that the majority of LNPs (blue) remain bound to the anionic SLB, while more than 80% of single-mRNA molecules (red) detach from the SLB at pH 7.5. See the main text for interpretation of these results. Scale bar 5 µm.

SUPPLEMENTARY TABLE

Table S1. Lipid compositions and characteristics of LNP formulations used in the presented study. mRNA encapsulation and concentration were determined using the RiboGreen assay. Size and concentration characterization were conducted using dynamic light scattering (DLS) and nanoparticle tracking analysis (NTA).

	low-DSPC LNPs	high-DSPC LNPs	calcein LNPs
Composition (lipids, mol%)			
DLin-MC3-DMA	53.47	50	53.47
DSPC	4.65	10	4.65
Chol	41.114	39.684	41.174
DSPE-PEG(2000) Biotin	0.006	0.006	0.006
DMPE-PEG(2000)	0.7	0.25	0.7
Rhod-DOPE	0.06	0.06	NA
Characteristics			
mRNA encapsulation (%)	97	98	34**
mRNA concentration (mg mL^{-1})	0.053	0.051	0.17
LNP diameter (nm)	139	142	145.5
LNP concentration (particles mL^{-1})*	1.26×10^{12}	0.82×10^{12}	2.20×10^{12}
PDI	0.031	0.019	<0.5*

*Measured by NTA

** PolyA

References

1. Agnarsson, B. *et al.* Low-temperature fabrication and characterization of a symmetric hybrid organic-inorganic slab waveguide for evanescent light microscopy. *Nano Futures* **2**, (2018).
